# Supplementary material for: Cancer patient survival can be parametrized to improve trial precision and reveal time-dependent therapeutic effects
Source: Nat Commun. 2022 Feb 15;13:873. doi: 10.1038/s41467-022-28410-9 (PMC8847344; doi:10.1038/s41467-022-28410-9)
Supplement: Supplementary file 5 — Supplementary Dataset 2 [file 41467_2022_28410_MOESM5_ESM.zip › Supplementary Data File 2/Small sample size calculations- Fig 4, S5, S6/12-month CIs/Precisionk10_CI_03052021.pdf]

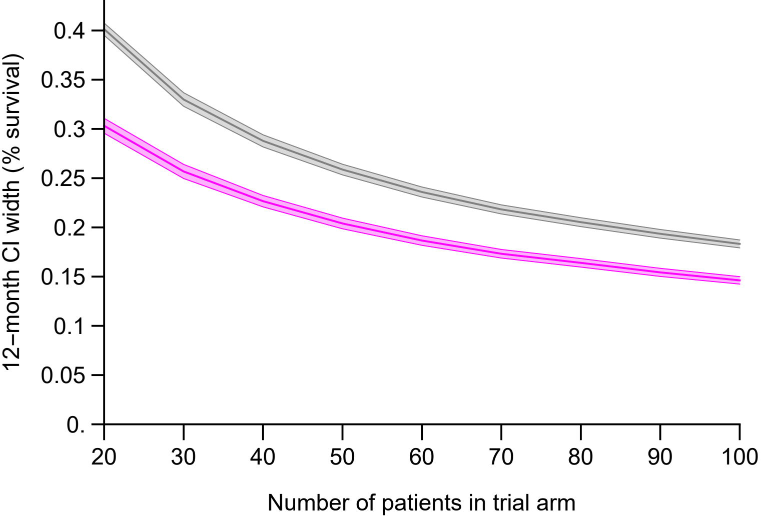

12–month CI width (% survival)
0.4
0.35
0.3
0.25
0.2
0.15
0.1
0.05
0.
20
30
40
50
60
70
80
90
100
Number of patients in trial arm
